# Patterns of oxytocin use for induction and augmentation of labour among healthcare providers in Nigeria

**DOI:** 10.1186/s12884-024-06593-x

**Published:** 2024-06-01

**Authors:** Chioma S. Ejekam, Ifeoma P. Okafor, Kehinde S. Okunade, Uchenna Igbokwe, Jude Nwokike

**Affiliations:** 1 Advanced Centre of Excellence of Drug Research, Herbal Medicine Development and Regulatory Science (ACEDHARS), University of Lagos, Akoka, Lagos Nigeria; 2 Department of Community Health and Primary Care, College of Medicine, University of Lagos, Idiaraba Lagos, Nigeria; 3 Department of Obstetrics and Gynaecology, College of Medicine, University of Lagos, Idiaraba Lagos, Nigeria; 4 Solina Centre for International Development and Research, Libreville Street, Wuse II, Abuja, Nigeria; 5 Promoting the Quality of Medicines Program Plus, U.S Pharmacopeia, Twinbrook Rockville, MD USA

**Keywords:** Patterns of oxytocin use, Intrapartum oxytocin use, Induction of labour, Augmentation of labour, Healthcare providers, Nigeria

## Abstract

**Background:**

The practice of intrapartum use of oxytocin for induction and augmentation of labour is increasing worldwide with documented wide variations in clinical use, especially dose administrations. There is also evidence of intrapartum use by unauthorized cadre of staff.

**Aim:**

This study assessed the patterns – frequency of intrapartum use of oxytocin, the doses and routes of administration for induction and augmentation of labour, and identified the predictors of oxytocin use for induction and augmentation of labour by healthcare providers in Nigeria.

**Methods:**

This was a cross-sectional study conducted among healthcare providers – doctors, nurses/midwives and community health workers (CHWs) in public and private healthcare facilities across the country’s six geopolitical zones. A multistage sampling technique was used to select 6,299 eligible healthcare providers who use oxytocin for pregnant women during labour and delivery. A self-administered questionnaire was used to collect relevant data and analysed using STATA 17 statistical software. Summary and inferential statistics were done and further analyses using multivariable regression models were performed to ascertain independent predictor variables of correct patterns of intrapartum oxytocin usage. The p-value was set at < 0.05.

**Results:**

Of the 6299 respondents who participated in the study, 1179 (18.7%), 3362 (53.4%), and 1758 (27.9%) were doctors, nurses/midwives and CHWs, respectively. Among the respondents, 4200 (66.7%) use oxytocin for augmentation of labour while 3314 (52.6%) use it for induction of labour. Of the 1758 CHWs, 37.8% and 49% use oxytocin for induction and augmentation of labour, respectively. About 10% of the respondents who use oxytocin for the induction or augmentation of labour incorrectly use the intramuscular route of administration and about 8% incorrectly use intravenous push. Being a doctor, and a healthcare provider from government health facilities were independent positive predictors of the administration of correct dose oxytocin for induction and augmentation of labour. The CHWs were most likely to use the wrong route and dose administration of oxytocin for the induction and augmentation of labour.

**Conclusion:**

Our study unveiled a concerning clinical practice of intrapartum oxytocin use by healthcare providers in Nigeria – prevalence of intrapartum use of oxytocin, inappropriate routes of administration for induction and augmentation of labour, varied and inappropriately high start dose of administration including unauthorized and high intrapartum use of oxytocin among CHWs.

**Supplementary Information:**

The online version contains supplementary material available at 10.1186/s12884-024-06593-x.

## Introduction

In recent times, evidence shows that more women undergo intrapartum use of oxytocin for induction and augmentation of labour, to deliver babies in the quest to shorten the duration of labour [1–3]. The World Health Organization recommends oxytocin, a uterotonic as a pharmacologic option for induction and augmentation of labour [4,5]. Induction of labour is one of the most common obstetric procedures and is performed in approximately 25% of all pregnancies in both high-income and low-middle-income countries [5,6]. It is indicated when the risks of continuing the pregnancy outweigh the risks of induction, with the goal of delivery while minimizing risks to the mother and newborn [7,8]. Induction is usually performed by administering intravenous (IV) oxytocin or prostaglandins or the manual rupturing of amniotic membrane to prevent the adverse outcomes associated with prolonged pregnancy [6–8]. Augmentation of labour is used to accelerate labour, shorten the time to delivery, and perhaps reduce the risk of caesarean delivery, particularly in women with longer labour or less frequent contractions [2,4,9]. It involves the stimulation of the uterus to increase the frequency, duration and intensity of contractions after the onset of labour to prevent delay in the first stage of labour [4]. Generally, labour augmentation has been done with intravenous infusion infusion of oxytocin and/or amniotomy [4]. 

Oxytocin when used for labour induction/augmentation can cause both maternal and foetal adverse outcomes [2,10], hence the need for strict adherence to recommendations and adequate provision for close monitoring [4,7]. Oxytocin used during labour has been associated with increased risks of neonatal encephalopathy [11], and increases the volume of blood loss during vaginal birth [12]. A Cochrane systematic review has shown that oxytocin used during labour has poor efficacy in reducing rates of caesarean section, and instrumental deliveries and is associated with uterine hyperstimulation and foetal heart rate changes [11,13]. 

In spontaneous labour, endogenous oxytocin release has been observed to follow a pulsatile pattern, with increasing pulse frequency, and short-lasting peaks start to occur at term to a maximum of three pulses/10 minutes [14]. However, synthetic oxytocin that is infused follows a flat pattern of release causing a different pattern of uterine contractions from that of physiological labour leading to irregular, more frequent, longer, and more painful contractions [14]. With oxytocin infusion at a dose of 0.6 International units/hour (IU/hour), the plasma level falls within the range of oxytocin level in physiological birth, but infusions influence the plasma levels in a dose-dependent pattern and doubling the dose results in doubling of the oxytocin levels and associated side effects unlike in physiological labour [15,16]. 

Wide variations in start dose administrations, interval and frequency of dose increase [14,17] have also been documented with this increasing practice of induction and augmentation of labour [2,3]. A review of regimens used in 12 countries reported an 11-fold difference with the total amount of International unit – IU of oxytocin infused ranging from 2.38 IU to 27.00 IU [14]. There is currently no national practice guideline on the intrapartum use of oxytocin in Nigeria, however, a low start dose of intrapartum oxytocin (2.5 IU up to 5 IU) with adequate caution in multiparous women is recommended based on experiences and guidelines from the different developed countries [18–21]. Global recommendations have called for cautious use of intrapartum oxytocin in multigravidas and women with a previous history of caesarean due to increased risk of uterine hyperstimulation and rupture [16,22,23]. 

Oxytocin as a high-alert medication bears a high risk of harm and hence requires specific evidence-based guidelines to reduce the likelihood of maternal and foetal harm [24]. Recommendations suggest the restriction to elective use of oxytocin, decreasing dosages when indicated, and granting more responsibility and authority for patient’s safety to the bedside professional staff [24]. Recent recommendations for oxytocin medication safety practices by the Institute for Safe Medication Practices further emphasized strict caution with its use [25]. The World Health Organization has cautioned the use of oxytocin in induction and augmentation of labour since the procedures carry the risks of complications, hence, should only be carried out in facilities where there is capacity to manage its potential adverse outcomes specifically, facilities with resources for assessing maternal and fetal well-being and where caesarean section can be performed immediately when needed [7]. Despite recommendations for the restricted use of intrapartum oxytocin, health professionals do not usually adhere to them. In low- and middle-income countries, initiation rates as high as 97% have been recorded in both primiparas and multigravidas and the majority of recipients (parturients) did not meet the criteria for prolonged labour [2]. Currently, nurses/midwives and doctors are trained with their respective training curricula in Nigeria in the use of oxytocin in labour while lower cadre birth attendants, including community health workers, are not allowed and potentially not trained in intrapartum use of oxytocin. In addition to having a longer clinical exposure and experience in the intrapartum and postpartum use of oxytocin, doctors also have the opportunity for frequently sponsored training and continuous medical education to improve their skills. Although the Nigeria national task shifting and sharing policy of 2014 permits CHWs to administer oxytocin for the prevention and initiation of treatment for postpartum haemorrhage, it does not approve this cadre of healthcare providers to administer the drug during labour for example, management of prolonged labour [26]. 

There is currently no accessible published data on practices regarding intrapartum oxytocin use in Nigeria. This paper forms part of a broader nationwide study that assessed the clinical experiences and use of oxytocin by healthcare providers who take deliveries in Nigeria. An aspect of the study that focused on the use of oxytocin for the prevention of post-partum haemorrhage among the study population has been published [27]. This second aspect described the patterns of intrapartum oxytocin injection use in induction and augmentation of labour by healthcare providers who deliver babies in Nigeria.

## Materials and methods

### Study design, site and population

This was a descriptive, cross-sectional survey conducted in early 2019 across Nigeria. Nigeria has 36 states (sub-regional government) and 774 local government areas organized into six geopolitical zones.

The study population consisted of healthcare providers in public (government) or private facilities that offer obstetric and gynaecological services and use oxytocin in their practices. Eligible study participants were healthcare providers (doctors, nurses/midwives, and community health workers) who provide intrapartum care in their practices and who consented to participate in this study. The community health workers were either community health officers or community health extension workers. Enrolment of the respondents – doctors, nurse/midwife, CHW in the study was based on the assumptions that each respondent who was seen and worked at the health facilities selected for this study were licensed to practice in Nigeria. Traditional birth attendants were excluded, as they are not recognized as part of the formal health care workforce in Nigeria.

### Sample size determination and sampling

The minimum sample size of 488 for each of the 12 states was determined using Cochran’s formula [28] and was based on 5% margin of error, 95% confidence interval, proportion (p) of 42.1% oxytocin storage practice from a previous study in Nigeria [29], and 30% addition to compensate for non-response to a self-administered questionnaire and recording errors. The minimum expected sample size was 5,856 in 12 states. In states where the allotted sample size could not be met, more respondents were selected from the other selected states within the same geo-political zone.

Respondents were selected using a multistage sampling technique. The first stage was the random selection of two states from each of the six geo-political zones using simple balloting, making for a total of 12 states. Simple balloting was also used to randomly select four local government areas from a list of all local government areas from each of the 12 states in the second stage. The third stage was selection of the public and private health facilities. All public healthcare facilities in the three tiers of health care namely, tertiary, secondary/general hospital and primary health centres that offer obstetrics and gynaecological services in the selected local government areas were included for recruiting respondents from the public healthcare sector. Using the list of registered private hospitals per local government areas from the State Ministries of Health, registered private healthcare facilities that offer obstetrics and gynaecology services were selected by systematic sampling per local government areas. If any selected health facility no longer exists or does not offer gynaecological services, the next one was selected. At the end of this stage, 1,894 healthcare facilities were selected. Finally, the selection of study respondents was based on the relative number of healthcare providers who met the inclusion criteria per facility. The doctor-to-nurse/midwife-to-CHW ratio of 1:2:1 was used to consecutively select respondents per facility. Two CHWs were selected in each of the health facilities where there were no doctors or nurses. In states with fewer healthcare facilities from which respondents would be selected, a maximum of six respondents were selected per facility in large public healthcare facilities. The final total sample of 6,299 healthcare providers, consisting of doctors, nurses/midwives and CHWs across Nigeria participated in the study.

### Data collection

Data was collected using the pretested structured self-administered questionnaire adapted from a previous similar study done in Lagos Nigeria [29]. The questionnaire included single and multiple-response questions aimed to capture respondents’ perspectives and different ways oxytocin was used in their clinical practice. The questionnaire had three sections. Section A collected information on respondents’ sociodemographic characteristics, section B assessed occupational history, and section C assessed clinical experience with oxytocin use. The questionnaire was pretested among 120 clinicians (doctors and nurses/midwives) who met the inclusion criteria, showed a willingness to participate, and were from facilities in the states not included in the study. The necessary corrections and feedback were used to improve the tool for clarity.

### Outcome measure

The primary outcomes of this study were the proportions of healthcare providers who use oxytocin for induction and/or augmentation of labour, the oxytocin dose and routes of administration used by healthcare providers for the induction and/or augmentation of labour including the correct dose of administration. For induction labour, the correct dosage was (5IU) and any other unit of dosage was considered as incorrect dosage. For augmentation labour, the correct dosage was (2.5IU or 5IU) and any other unit of dosage was considered incorrect dosage. (Appendix 1) The covariates were professional cadre of respondents, type of facility, and years of work experience in their current role.

### Data analysis

Data analyses were done using the STATA 17. Univariate, bivariate, and multivariate analyses were used to assess the relationship between the outcome variables and covariates. At the univariate level, categorical variables were presented in frequency tables and proportions while means and standard deviations were estimated for continuous variables. In bivariate analysis, Pearson’s chi-square analysis was used to test for association. We conducted a binary logistic regression model and only predictor variables that were statistically significant were included in the multivariable logistic regression model. The multivariable logistic regression modelling was conducted on the dosage of oxytocin administration for induction and augmentation of labour in primigravidae and multigravidas using significant variables. Statistical significance was set at *p* < .05 for all analyses. Results were presented in adjusted odds ratios and 95% confidence intervals.

## Results

There were 6299 respondents who participated in the study. Out of these, 1179 (18.7%), 3362 (53.4%), and 1758 (27.9%) of them were doctors, nurses/midwives and community health workers, respectively. About 59% of the respondents were ≤ 10 years of practice in their profession and 52% of the respondents were from government (public) healthcare facilities. Of the enrolled respondents, 52.6% use oxytocin for the induction of labour while 66.7% of them use it for augmentation of labour. (Table 1)

**Table 1:** Socio-demographic characteristics and clinical variable of study respondents (*n* = 6299)

Variable	Category	Frequency	Percentage
Age group	<=34 years	2759	43.8
	35–54 years	3181	50.5
	> 54 years	359	5.7
*Mean ± SD*	***37.6 ± 10.2***		
Sex	Male	1394	22.1
	Female	4905	77.9
Profession	Doctor	1179	18.7
	Nurse/midwife	3362	53.4
	Community Health Worker (CHW)	1758	27.9
Years of practice	≤ 10 years	3711	58.9
	> 10 years	2588	41.1
Health facility of practice	Government	3274	52.0
	Private	3025	48.0
Indications for use of oxytocin*	Prevention of postpartum hemorrhage (PPH)	4908	77.9
	Induction of labor	3314	52.6
	Augmentation of labor	4200	66.7

*multiple response SD – Standard deviation

**Fig. 1:**
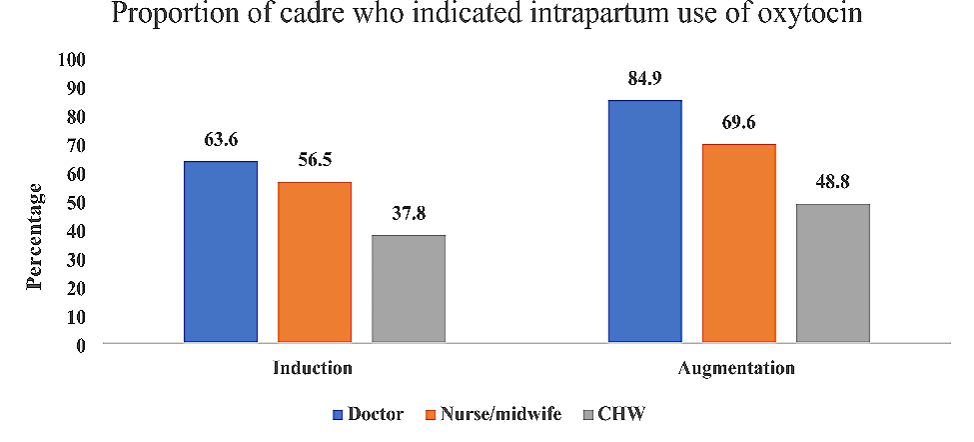
Proportion of healthcare providers’ cadre (out of *n* = 1179 doctors, *n* = 3362 nurses/midwives, and *n* = 1758 CHWs) who indicated intrapartum use of oxytocin

Of the 1179 doctors who participated in the larger study, 63.6% used oxytocin for induction of labour and 84.9% used oxytocin for augmentation of labour. Of the 3362 nurses/midwives who were part of this study, 56.5% used oxytocin for induction of labour while 69.6% used it for augmentation of labour. Of the 1758 CHWs who participated, 37.8% used oxytocin for induction of labour while 48.8% used it for the augmentation of labour. (Fig. 1).

Table 2 shows that of the 3314 respondents who indicated the use of oxytocin for induction of labour, about 47.7% of them practice in government health facilities and 52.3 were from private health facilities. Likewise, 48.4% of the 4200 respondents who indicated the use of oxytocin for augmentation of labour were from the government health facilities. Similar proportions of the respondents who indicated the use of oxytocin for the induction(78.9) or augmentation of labour(78.0) reported that they have had previous training (pre- or in-service) on the use. The majority of the respondents who used intrapartum oxytocin for their clients administered it through the intravenous route. As much as 10.3% and 9.4% used intramuscular routes to administer oxytocin for induction of labour and augmentation of labour respectively while about 8% reported using intravenous push to administer oxytocin for the induction and augmentation of labour.

**Table 2:** Respondents’ characteristics and intrapartum use of oxytocin

Variable	Category	Induction (*n* = 3314) Freq. (%)	Augmentation (*n* = 4200) Freq. (%)
Health facility of practice	Government	1580 (47.7)	2033 (48.4)
	Private	1734 (52.3)	2167 (51.6)
Profession	Doctors	750 (22.6)	1001 (23.8)
	Nurse/midwife	1899 (57.3)	2341 (55.7)
	CHW	665 (20.1)	858 (20.4)
Previous training on use oxytocin	Yes	2616 (78.9)	3274 (78.0)
	No	606 (18.3)	786 (18.7)
	Not sure	92 (2.8)	139 (3.3)
Respondents route of administration for intrapartum oxytocin use *	Intramuscular	341(10.3)	393(9.4)
	Intravenous push	262(7.9)	315(7.5)
	Intravenous infusion	3053(92.1)	3879(92.4)
	Other	80 (2.4)	90 (2.1)

multiple response*

**Fig. 2:**
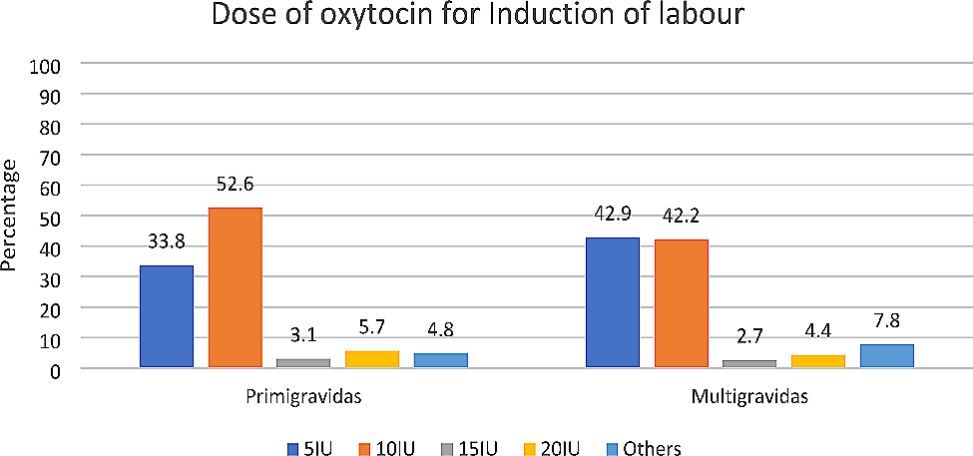
Dose of oxytocin used for the induction of labour in primigravid and multigravid women (*n* = 3314)

The respondents (*n* = 3314) were asked about the dose of administration of oxytocin used for the induction in primigravida and multigravida. Just over half (52.6%) used 10IU of oxytocin for induction of labour in primigravidae while 42.2% used 10IU in multigravidas. Up to 33.8% and 42.9% used 5IU of oxytocin for induction of labour in primigravidae and multigravidas respectively while 5.7% and 4.4% used 20IU for induction of labour in primigravids and multigravids respectively. (Fig. 2)

**Fig. 3:**
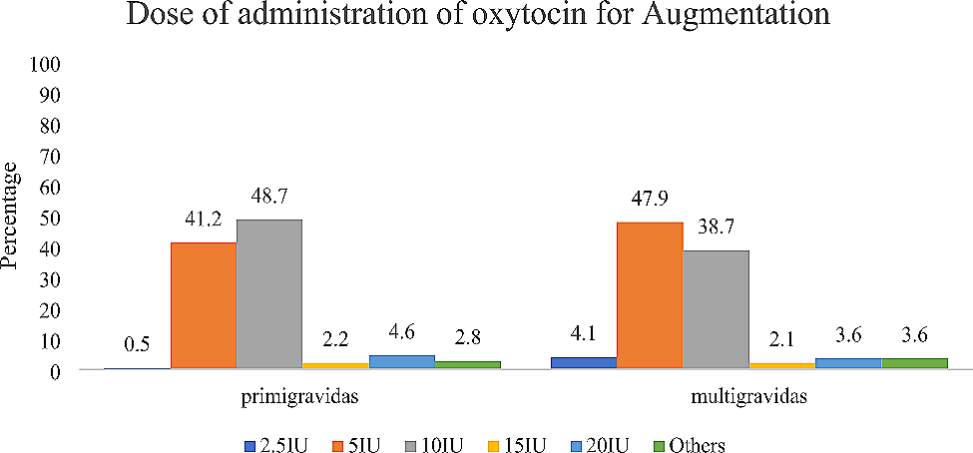
Dose of oxytocin used for augmentation of labour in primigravid and multigravida women (*n* = 4200)

Figure 3 shows that 48.7% and 38.7% of the respondents (*n* = 4200) used 10IU of oxytocin for the augmentation of labour in primigravids and multigravidas respectively while 4.6% and 3.6% used 20IU of oxytocin for the augmentation of labour in primigravids and multigravidas respectively.

Table 3 shows that a higher proportion of respondents who were CHWs used the intramuscular route for administering oxytocin for the induction of labour and augmentation of labour compared to respondents who were doctors (*p* < .001).

**Table 3:** Oxytocin routes of administration for the induction and augmentation of labour by covariates

**Oxytocin induction route of administration by profession, years of practice and health facility of practice (** ***n*** ** = 3314)**							
**Variable**	**Category**	**Intramuscular**	**Intravenous push**	**Intravenous infusion**	**Other**	**Total**	**X** ^**2**^ **(** ***P*** **-Value)**
Profession*	Doctor(n-750)	55 (6.5)	65 (7.7)	709 (84.3)	12 (1.4)	841	107.7(< 0.001)
	Nurse/Midwife (n-1899)	164 (7.7)	135 (6.3)	1768 (82.7)	70 (3.3)	2137	
	CHW(n-665)	122 (15.6)	62 (7.9)	576 (73.7)	22 (2.8)	782	
Years of practice	≤ 10 years	224 (10.1)	148 (6.7)	1774 (80.4)	61 (2.8)	2207	9.7 (0.047)
	> 10 years	117 (7.5)	114 (7.3)	1279 (82.4)	43 (2.8)	1553	
Health facility of practice	Government	161 (8.9)	127 (7.1)	1453 (80.9)	54 (3.0)	1795	0.99 (0.91)
	Private	180 (9.2)	135 (6.9)	1600 (81.4)	50 (2.5)	1965	
**Oxytocin augmentation route of administration by profession, years of practice and health facility of practice** (*n = 4200)*							
**Variable**	**Category**	**Intramuscular**	**Intravenous push**	**Intravenous infusion**	**Other**	**Total**	**X** ^2^ **(P-Value)**
Profession *	Doctor (n-1001)	52 (4.8)	83 (7.6)	948 (86.7)	11 (1.0)	1094	239.1(< 0.001)
	Nurse/Midwife (n-2341)	175 (6.8)	148 (5.7)	2202 (85.5)	50 (1.9)	2575	
	CHW(n-858)	166 (16.5)	84 (8.3)	729 (72.4)	28 (2.7)	1007	
Years of practice*	≤ 10 years	253 (8.9)	189 (6.7)	2317 (82.4)	54 (2.0)	2813	5.5 (0.24)
	> 10 years	140 (7.5)	126 (6.8)	1562 (83.8)	35 (1.9)	1863	
Health facility of practice*	Government	217 (9.5)	147 (6.4)	1880 (82.3)	39 (1.7)	2283	9.5 (0.05)
	Private	176 (7.4)	168 (7.0)	1999 (83.5)	50 (2.0)	2393	

*multiple response table

In Table 4, the bivariate analysis showed that doctors were the highest proportion of healthcare providers to use 5IU for the induction of labour in primigravidae while CHWs were the highest proportion who used 20IU of oxytocin for the induction of labour in primigravidae (*p* < .05). A higher proportion of healthcare providers from the private healthcare facilities reported use of 20IU starting dose for induction of labour among primigravids than the respondents from the government healthcare facilities (*p* < .001). Similarly in the multigravidas, the bivariate analysis showed that the CHWs were most associated with the use of 20IU for the induction of labour in multigravidas. This was statistically significant (*p* < .001). Higher proportions of respondents from the private healthcare facilities reported the use of a 20IU starting dose in the induction of labour among multigravidas than the respondents from the government healthcare facilities and this difference was also statistically significant (*p* < .01).

**Table 4:** Respondents’ profession, years of practice and facility type by oxytocin doses of administration for the induction of labour in primigravids and multigravidas

**Variable**	**Category**	**5IU**	**10IU**	**15IU**	**20IU**	**Other**	**Total**	**X** ^2^ **(P-Value)**
Profession	Doctor(n-750)	274 (36.5)	390 (52.0)	22 (2.9)	34 (4.5)	30 (4.0)	750	17.2 (0.03)
	Nurse/Midwife (n-1899)	638 (33.6)	1012 (53.3)	57 (3.0)	98 (5.2)	94 (4.9)	1899	
	CHW(n-665)	207 (31.1)	342 (51.4)	24 (3.6)	56 (8.4)	36 (5.4)	665	
Years of practice	≤ 10 years	679 (35.1)	986 (51.0)	59 (3.1)	104 (5.4)	105(5.4)	1933	9.1 (0.06)
	> 10 years	440 (31.9)	758 (54.9)	44 (3.2)	84 (6.1)	55 (4.0)	1381	
Facility type	Government	565 (35.8)	791 (50.1)	57 (3.6)	78 (4.9)	89 (5.6)	1580	16.7 (0.002)
	Private	554 (31.9)	953 (55.0)	46 (2.7)	110 (6.3)	71 (4.1)	1734	
**Oxytocin dose of administration used for induction of labour in multigravida women by profession, years of practice and health facility of practice** (*n = 3314)*								
**Variable**	**Category**	**5IU**	**10IU**	**15IU**	**20IU**	**Other**	**Total** **(n)**	**X** ^2^ **(P-Value)**
Profession	Doctor(n-750)	357(47.6)	290(38.7)	21(2.8)	22(2.9)	60(8.0)	750	36.2(< 0.001)
	Nurse/Midwife (n-1899)	834(43.9)	780(41.1)	44(2.3)	89(4.7)	152(8.0)	1899	
	CHW(n-665)	231(34.7)	327(49.2)	26(3.9)	36(5.4)	45(6.8)	665	
Years of practice	≤ 10 years	801(42.4)	818(42.3)	55(2.9)	96(5.0)	163(8.4)	1933	8.2(0.08)
	> 10 years	621(45.0)	579(41.9)	36(2.6)	51(3.7)	94(6.8)	1381	
Health facility of practice	Government	742(47.0)	602(38.1)	49(3.1)	50(3.2)	137(8.7)	1580	39.0(< 0.001)
	Private	680(39.2)	795(45.9)	42(2.4)	97(5.6)	120(7.0)	1734	

In the multivariable logic regression, (Table 5), the most significant predictor of using the correct dose (5IU) of oxytocin for induction of labour in both primigravidae and multigravidas was being a doctor. The doctors were 1.38 times (95% CI: 1.10–1.73) more likely to use the correct dose (5IU) of oxytocin for induction of labour in primigravida and 1.94 times (95% Cl:1.56–2.43) in multigravida women respectively compared to CHWs. Nurses/ midwives were 1.60 times (95% Cl, 1.32–1.92) more likely to use the correct doses of oxytocin for induction of labour in multigravida women compared to CHWs. Healthcare providers who have more than 10 years of working experience were 1.22 times (95% Cl, 1.05–1.42) more likely to use the correct dose of oxytocin for induction of labour in primigravida compared to those with less than 10 years of working experience. Healthcare providers from the government health facilities were 1.29 times (95% Cl, 1.11–1.50) more likely to use the correct dose of oxytocin for induction of labour in primigravida and 1.49 times (95% Cl, 1.29–1.72) in multigravida women more than those from private health facilities.

**Table 5:** Independent predictors of correct doses of oxytocin for induction of labour in both Primigravid and Multigravid women (*n* = 3314)

Predictors	Categories	Primigravida	Multigravida
		(AOR) [95% CI]	(AOR) [95% CI]
Profession	CHW Doctor Nurse/midwife	Ref. 1.38 (1.10–1.73)* 1.17 (0.96–1.41)	Ref. 1.94 (1.56–2.43)* 1.60 (1.32–1.92)*
Years of experience	≤ 10years > 10years	Ref. 1.22 (1.05–1.42)*	Ref. 0.93 (0.81–1.08)
Health facility of practice	Private Government	Ref. 1.29 (1.11–1.50)*	Ref. 1.49 (1.29–1.72)*

*statistically significant

Table 6 shows a bivariate analysis of the doses of oxytocin used by respondents for the augmentation of labour in primigravids and the covariates. The CHWs have the highest proportion of those who use 20IU and the least proportion to use 5IU starting dose for the augmentation of labour in primigravids and this difference was statistically significant (*p* < .05). Similarly in the multigravidas, doctors were more likely to use 2.5IU starting dose than CHWs for the augmentation of labour (*p* < .001). A higher proportion of respondents from the government healthcare facilities use 2.5IU starting dose in the augmentation of labour among multigravidas compared to respondents from the private healthcare facilities (*p* < .001).

**Table 6:** Respondents’ profession, years of practice and facility type by oxytocin doses of administration for the augmentation of labour in primigravid and multigravida women (*n* = 4200)

**Variable**	**2.5IU**	**5IU**	**10IU**	**15IU**	**20IU**	**Other**	**Total(n)**	**X** ^2^ **(P-Value)**
**Profession**								
Doctor (n-1001)	6(0.6)	248(42.8)	492 (49.2)	16 (1.6)	39 (3.9)	20 (2.0)	1001	21.0(0.021)
Nurse/ Midwife (n-2341)	18 (0.8)	993 (47.5)	1112(47.5)	55 (2.4)	106(4.5)	57 (2.4)	2341	
CHW (n-858)	6(0.7)	308(35.9)	443(51.6)	20 (2.3)	49(5.7)	32(3.7)	858	
**Years of practice**								
≤ 10 years	15(0.6)	1036(41.1)	1220(48.4)	54(2.1)	127(5.0)	68(2.7)	2520	4.1 (0.54)
> 10 years	15(0.9)	693(41.3)	827(49.2)	37(2.2)	67(4.0)	41(2.4)	1680	
**Health facility of practice**								
Government	20(1.0)	846(41.6)	976(48.0)	44(2.2)	86(4.2)	61(3.0)	2033	8.4 (0.14)
Private	10(0.5)	883(40.8)	1071(49.4)	47(2.2)	108(5.0)	48(2.2)	2167	
**Oxytocin dose of administration used for augmentation of labour in multigravidas by profession, years of practice and health facility of practice**								
**Variable**	**2.5IU**	**5IU**	**10IU**	**15IU**	**20IU**	**Other**	**Total(n)**	**X** ^2^ **(P-Value)**
**Profession**								
Doctor (n-1001)	554 (55.3)	329 (32.9)	12 (1.2)	18 (1.8)	67 (6.7)	21 (2.1)	1001	123.7 (< 0.001)
Nurse/ Midwife (n-2341)	1132 (48.4)	904 (38.6)	57 (2.4)	76 (3.3)	87 (3.7)	85 (3.6)	2341	
CHW (n-858)	326 (38.0)	397 (46.3)	19 (2.2)	55 (6.4)	17 (2.0)	44 (5.1)	858	
**Years of practice**								
≤ 10 years	1168 (46.4)	1000 (39.7)	51 (2.0)	93 (3.7)	104 (4.1)	104 (4.1)	2520	10.4 (0.06)
> 10 years	844 (50.2)	630 (37.5)	37 (2.2)	56 (3.3)	67 (3.7)	46 (2.7)	1680	
**Health facility of practice**								
Government	1039 (51.1)	707 (34.8)	47 (2.3)	66 (3.3)	99 (4.9)	75 (3.7)	2033	33.2 (< 0.001)
Private	973 (44.9)	923 (42.6)	41 (1.9)	83 (3.8)	72 (3.3)	75 (3.5)	2167	

The most significant predictor of using the correct dose (2.5IU − 5IU) of oxytocin for augmentation of labour in both primigravida and multigravidas was being a doctor. The doctors were 1.38 times (95%CI: 1.14–1.67) more likely to use the correct dose of oxytocin for augmentation of labour in primigravida and 1.34 times (95%Cl: 1.02–1.77) in multigravida women compared to CHWs and Nurses/ midwives were 1.35 times (95% Cl: 1.14–1.59) more likely to use the correct doses of oxytocin for augmentation of labour in primigravid women compared to CHWs (Table 7).

**Table 7:** Independent predictors of correct doses of oxytocin for augmentation of labour in both Primigravid and Multigravid women (*n* = 4200)

Predictors	Categories	Primigravida	Multigravida
		(AOR) [95% CI]	(AOR) [95% CI]
Profession	CHW Doctor Nurse/midwife	Ref. 1.38 (1.14–1.67)* 1.35 (1.14– 1.59)*	Ref. 1.34 (1.02–1.77) 1.22 (0.98–1.52)
Years of experience	≤ 10years > 10years	Ref. 1.0 (0.88– 1.14)	Ref. 0.83 (0.69–1.01)
Health facility of practice	Private Government	Ref 1.12 (0.98–1.27)	Ref. 0.88 (0.73–1.06)

*statistically significant

## Discussion

The frequencies of intrapartum oxytocin use among respondents in this study (52.6%-induction and 66.7%-augmentation of labour) were lower than that of a Lagos study conducted in 2017 in which 67.9% and 80.0% of healthcare providers used oxytocin for induction and augmentation of labour respectively [29]. Yet the Lagos study and the current finding have similar patterns, in that oxytocin was used more for augmentation of labour than for induction of labour [29]. Previous studies have reported that oxytocin was used for augmentation of labour in 37.0% of deliveries in a Nepal study [30] and labour induction in a United States in 2020 study was 31.4% [31]. This is a significant increase from the World Health Organization figure of 10.0% published from the data of 24 member countries over a decade ago [5]. This increasing use of intrapartum oxytocin globally [2,3,17,29] could suggest improvements in intrapartum feto-maternal monitoring [4] and neonatal resuscitation techniques following perinatal asphyxia [30,32]. . However, the relatively higher frequency of intrapartum oxytocin use reported in our study is concerning as the quality of intrapartum monitoring that characterises most resource-limited settings is still far from meeting the World Health Organization standards [33,34]. 

Our study found that in comparison to other categories of healthcare providers, the frequency of intrapartum oxytocin use for either induction or augmentation of labour was higher among doctors. This is not unusual, as doctors, due to a longer and more comprehensive training curriculum, are expected to have better skills in feto-maternal monitoring and early recognition of danger signs with a higher threshold for referral and reversion to caesarean delivery during induction and augmentation of labour [35]. Although the national task shifting and sharing policy permits lower cadre birth attendants such as CHWs to administer oxytocin for the prevention and initiation of treatment for postpartum haemorrhage only [26,29], there is evidence from our findings that this is not strictly adhered to, as many of them practice intrapartum oxytocin use (over a third use oxytocin for induction and nearly half for augmentation of labour). This is worrisome as CHWs are often present and work in primary health centres where the capacity to attend to potential obstetric complications and even caesarean section is lacking. The preparedness of a health facility to attend to surgical obstetric emergencies with requisite skills is necessary during the use of oxytocin in labour [5,36]. This finding is similar to previous reports of healthcare providers deviating from existing national guidelines on the intrapartum use of oxytocin [37] and knowledge gaps have been identified to be a potential contributor to this unsafe practice [38]. Furthermore, according to our results, oxytocin was incorrectly administered via the intramuscular route and intravenous push for induction and augmentation of labour. Being a CHW was also significantly associated with the practice of intramuscular administration of oxytocin for both induction and augmentation of labour.

Oxytocin is a potentially harmful medication and cautious use is recommended as risks may be higher in low-resource settings where the capacity to manage complications may be limited in most health facilities [2,30]. Expectedly, a major proportion of the healthcare providers claimed to have been previously trained in the use of oxytocin although the content and scope of this training are not ascertained for the different cadre of health providers. This should however be augmented by continuous training programs to ensure evidence-based practice and sustain the quality of care to be provided by these health care providers. In addition to continuous training and education, addressing the misuse of oxytocin by CHWs requires other multifaceted approaches including stricter regulation, community engagement, and supply chain management. By implementing these mitigation pathways, we can safeguard maternal and infant health while maintaining the essential role of CHWs in our fragile healthcare systems.

The World Health Organization recommends only oxytocin intravenous route of administration, especially via intravenous infusion for induction or augmentation of labour when indicated. The intramuscular route of oxytocin administration in labour is said to have a more sustained and longer-lasting uterine contraction of 30–60 min which could be dangerous to the woman and foetus. These findings underscore both the importance of developing a standardized evidence-based practice guideline for intrapartum oxytocin use in Nigeria [23] and the need to ensure continuous training for healthcare providers who attend to women during labour and childbirth while ensuring strict adherence to limitation of practice among the cadres of healthcare providers.

Most international guidelines and standard operating procedures have recommended a very low oxytocin start dose of 2.5IU or less and a maximum of 5IU [16,22,36]. However, our study found that significant proportions of the healthcare providers used 10IU of oxytocin for both induction and augmentation for primigravidae and multigravidae. This practice is contraindicated especially in multigravidae due to the increased risk of hyperstimulation and uterine rupture [3,13,16,22,23], and more importantly a deviation from most global guidelines on the intrapartum use of oxytocin [20,21,36]. 

Our findings showed that being a doctor was the most significant predictor for the use of the correct oxytocin dose for induction and augmentation of labour in both primigravidae and multigravidas. Interestingly, being a CHW was significantly associated with the use of a 20IU dose for the induction of labour in both primigravids and multigravids. This further highlights the urgent need for continuous in-service training on the use of oxytocin with appropriate content and scope, and to further reinforce the understanding of limitations in clinical practice as CHWs are not authorized to administer intrapartum oxytocin. These findings suggest the lack of a unified standard operating procedure for the use of oxytocin in labour within healthcare facilities even among members of the same team. It may also point to the possible use of low-quality and ineffective oxytocin which could have an influence on the administered dose by these healthcare providers to effectively stimulate uterine contractions. Evidence from previous studies conducted in similar resource-constrained settings [27,38,39] have reported that only a few healthcare providers have correct knowledge of the appropriate oxytocin storage which potentially affects storage practice and oxytocin quality, and healthcare providers often use doses beyond global recommendations to achieve desired contractions.

In addition, we found that having over 10 years of work experience and working in government-owned health facilities were positive independent predictors of the use of correct oxytocin dosage for induction of labour in primigravidae while working in a government-owned health facility also positively predicted correct oxytocin dose of administration in multigravidae. This may not be surprising as longer years of clinical exposure provide opportunities for training and re-training and in addition, the government-owned health facilities in Nigeria are expected to have more qualified healthcare providers due to stringent recruitment processes. Furthermore, these government-owned health facilities, compared to privately-owned health facilities, provide better opportunities for frequently sponsored training and continuous medical education to their healthcare providers.

There were a few limitations in this study. Firstly, the study outcomes were self-reported thus the possible introduction of bias in the accurate recall of respondents’ actual practice experience. Secondly, a qualitative study would have provided additional context necessary to complement the information provided in this study. However, this is the largest study that will add to the body of knowledge on healthcare providers’ patterns of intrapartum oxytocin use in Nigeria.

## Conclusion

Our study unveiled concerning practice of intrapartum oxytocin use by healthcare providers in Nigeria. We reported an inappropriate route of administration, the unauthorized intrapartum use of oxytocin among healthcare providers, especially CHWs, in addition to inappropriately high doses of oxytocin used for induction and augmentation of labour in women in Nigeria. We observed that being in the higher cadre of the health profession – especially being a doctor, having > 10 years of working experience and working in a government health facility were significant predictors of the use of correct oxytocin dosage for either induction or augmentation of labour. We, therefore, call for more cautious use of intrapartum oxytocin with clear indications, the need to develop a robust evidence-based practice guideline and ensure content and scope of training on intrapartum oxytocin including the appropriate methods of administration, initial effective dose, and the correct dose escalation rate, particularly in low-resource countries. There is a need for capacity building to bridge the healthcare providers’ knowledge and clinical practice gaps around the recommended route and dose of intrapartum oxytocin administration. Furthermore, there is a need for emphasis on strict definition of practice limitations for all cadre of healthcare staff.

### Electronic supplementary material

Below is the link to the electronic supplementary material.

Supplementary Material 1

## Data Availability

All data generated or analysed during this study are included in this published article.

## Abbreviations

CHOs: Community Health officers
CHWs: Community Health Workers
CHEWs: Community Health Extension Workers
IU: International units
IV: Intravenous; SD: Standard deviation
