# Supplementary material for: Patterns of oxytocin use for induction and augmentation of labour among healthcare providers in Nigeria
Source: BMC Pregnancy Childbirth. 2024 Jun 1;24:403. doi: 10.1186/s12884-024-06593-x (PMC11144308; doi:10.1186/s12884-024-06593-x)
Supplement: Supplementary file 1 — Supplementary Material 1 [file 12884_2024_6593_MOESM1_ESM.docx]

**Supplementary file**

**Appendix 1:**

**Categorization of oxytocin dose administration for induction and augmentation of labour**

| **Variable** | **Dosage for Induction** | | **Dosage for Augmentation** | |
| --- | --- | --- | --- | --- |
|  | **Primigravida** | **Multigravida** | **Primigravida** | **Multigravida** |
| **Correct dosage** | 1119 (33.77) | 1422 (42.91) | 1759 (41.9) | 3642 (86.71) |
| **Incorrect dosage** | 2195 (66.23) | 1892 (57.09) | 2441 (58.1) | 558 (13.29) |
